# Filling out the structural map of the NTF2-like superfamily

**DOI:** 10.1186/1471-2105-14-327

**Published:** 2013-11-19

**Authors:** Ruth Y Eberhardt, Yuanyuan Chang, Alex Bateman, Alexey G Murzin, Herbert L Axelrod, William C Hwang, L Aravind

**Affiliations:** 1 Wellcome Trust Sanger Institute, Wellcome Trust Genome Campus, Hinxton, Cambridgeshire, CB10 1SA, UK; 2 European Molecular Biology Laboratory, European Bioinformatics Institute, Hinxton, Cambridgeshire, CB10 1SD, UK; 3 Sanford Burnham Medical Research Institute, 10901 North Torrey Pines Road, La Jolla, CA, 92037, USA; 4 MRC Laboratory of Molecular Biology, Francis Crick Avenue, Cambridge Biomedical Campus, Cambridge, CB2 0QH, UK; 5 Stanford Synchrotron Radiation Lightsource, SLAC National Accelerator Laboratory, Menlo Park, CA, USA; 6 National Center for Biotechnology Information, NLM, NIH, Bethesda, MD, 20814, USA

**Keywords:** NTF2-like superfamily, Protein function prediction, Protein structure, Ligand-binding, JCSG, 3D structure, Protein family

## Abstract

**Background:**

The NTF2-like superfamily is a versatile group of protein domains sharing a common fold. The sequences of these domains are very diverse and they share no common sequence motif. These domains serve a range of different functions within the proteins in which they are found, including both catalytic and non-catalytic versions. Clues to the function of protein domains belonging to such a diverse superfamily can be gleaned from analysis of the proteins and organisms in which they are found.

**Results:**

Here we describe three protein domains of unknown function found mainly in bacteria: DUF3828, DUF3887 and DUF4878. Structures of representatives of each of these domains: BT_3511 from *Bacteroides thetaiotaomicron* (strain VPI-5482) [PDB:3KZT], Cj0202c from *Campylobacter jejuni* subsp. jejuni serotype O:2 (strain NCTC 11168) [PDB:3K7C], rumgna_01855) and RUMGNA_01855 from *Ruminococcus gnavus* (strain ATCC 29149) [PDB:4HYZ] have been solved by X-ray crystallography. All three domains are similar in structure and all belong to the NTF2-like superfamily. Although the function of these domains remains unknown at present, our analysis enables us to present a hypothesis concerning their role.

**Conclusions:**

Our analysis of these three protein domains suggests a potential non-catalytic ligand-binding role. This may regulate the activities of domains with which they are combined in the same polypeptide or via operonic linkages, such as signaling domains (e.g. serine/threonine protein kinase), peptidoglycan-processing hydrolases (e.g. NlpC/P60 peptidases) or nucleic acid binding domains (e.g. Zn-ribbons).

## Background

The NTF2-like superfamily is a large group of related proteins that share a common fold, first observed in the structure of the rat NTF2 (Nuclear Transport Factor 2) protein [1]. It is a versatile fold that can accommodate very different sequences and has no characteristic sequence motif associated with it. The NTF2-like fold has a cone-like shape with a cavity inside and acts as a molecular container that can be adapted to serve a broad range of different functions.

The NTF2-like proteins can be broadly defined into two functional categories: enzymatically active and non-enzymatically active proteins. The intracellular examples of this fold include most of the enzymatic functions associated with these proteins. These include SnoaL polyketide cyclase, scytalone dehydratase, limonene-1,2-epoxide hydrolase and δ5-3-ketosteroid isomerase [2-5]. The extracellular NTF2-like proteins tend to be non-enzymatic and possess small molecule binding activity. Non-enzymatic members of this superfamily include NTF2 [1], a domain found at the C-terminus of calcium/calmodulin dependent protein kinase II which is responsible for the multimerization of these kinases [6] and Mba1, a protein which binds to ribosomes and may function as a receptor [7]. NTF2-like domains have been found in proteins involved in bacterial conjugation where a multiprotein complex, the type IV secretion system, mediates transfer of plasmid DNA from a donor to a recipient bacterial cell [8-10]. More recently the non-catalytic NTF2-like domains have also been shown to function as immunity proteins in the bacterial polymorphic toxin systems [11].

Release 27.0 of the Pfam database [12] includes 24 different families as part of the NTF2 superfamily. Of these families, 21 have at least one representative where the three-dimensional structure has been deposited in the PDB. To date, the PDB contains at least 170 structures with NTF2-like fold, including at least 27 structures solved by the Joint Center for Structural Genomics (JCSG). Here we describe the first crystal structures of three Pfam families with NTF2-like folds: DUF3828 [PDB:3KZT] [Pfam:PF12883], DUF3887 [PDB:4HYZ] [Pfam:PF13026] and DUF4878 [PDB:3K7C] [Pfam:PF12870].

## Results and discussion

### Domain descriptions

DUF3828 family [Pfam: PF12883] is annotated in Pfam as a domain of unknown function. It is present in 492 different UniProtKB proteins from 451 different organisms. It is found exclusively in Gram-negative bacteria, with the vast majority of the species it occurs in belonging to the Enterobacteraceae family. [Pfam: PF12870] was previously annotated in Pfam as a lumazine-binding domain, however this has since been found to be incorrect and so we have renamed this family as a domain of unknown function, DUF4878. This domain is present in 650 different UniProtKB proteins from 571 different species. Like DUF3828, DUF4878 is a bacterial family, however it is found in a wider variety of bacterial species. It is found in both Gram-negative bacteria (including Proteobacteria and Bacteroidetes) and Gram-positive species (including Firmicutes and Actinobacteria). Finally, DUF3887 family [Pfam: PF13026] is another domain of unknown function. This domain is present in 364 different UniProtKB proteins from 262 different species. It is predominantly found in Firmicutes, but is also present in other phyla, including several Archaeal species.

**Figure 1:**
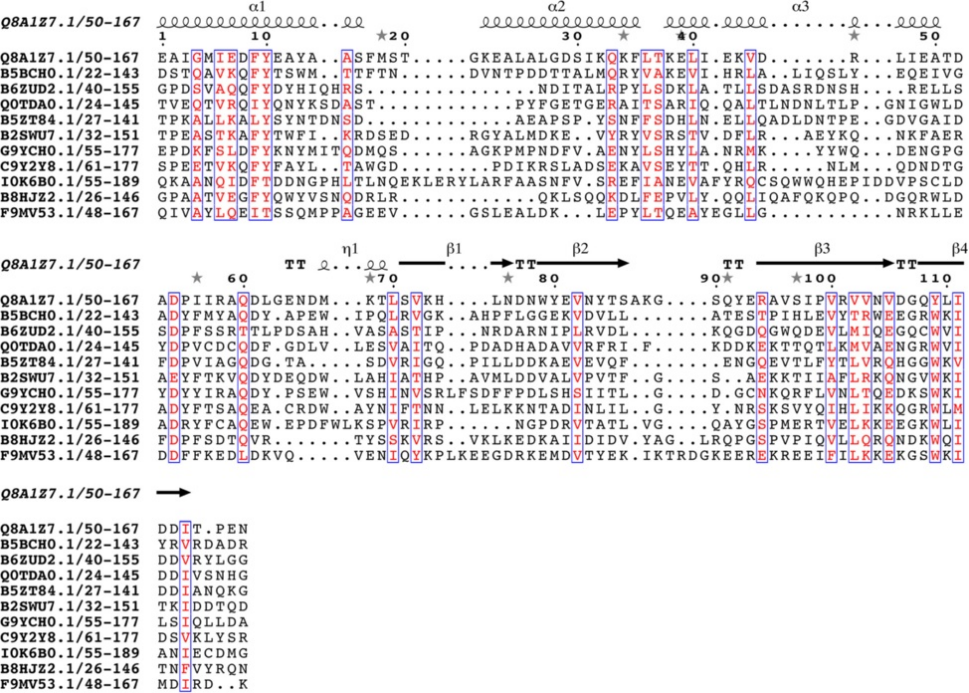
**Sequence alignment of DUF3828.** Conserved residues are highlighted in open red boxes. The secondary structure is shown above the alignment. The alignment was displayed using ESPript [13].

**Figure 2:**
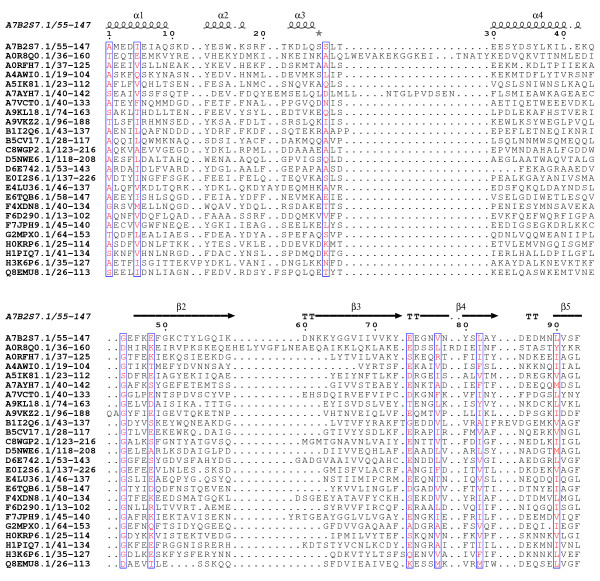
**Sequence alignment of DUF3887.** Conserved residues are highlighted in open red boxes. The secondary structure is shown above the alignment. The alignment was displayed using ESPript [13].

**Figure 3:**
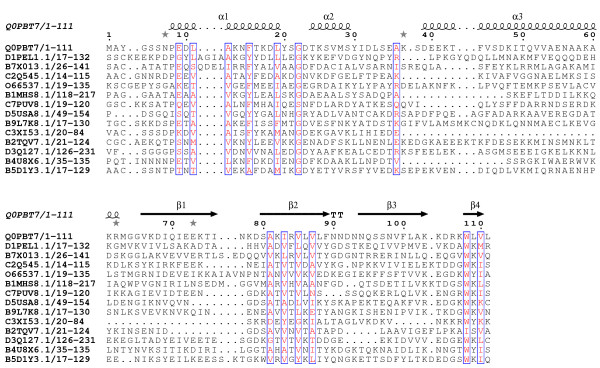
**Sequence alignment of DUF4878.** Conserved residues are highlighted in open red boxes. The secondary structure is shown above the alignment. The alignment was displayed using ESPript [13].

All three of these domains are of a similar length (around 100 amino acids). The N-terminus of DUF3828 (Figure 1) contains a pair of conserved aromatic amino acids (phenylalanine and tyrosine). There is a conserved aspartic acid in the middle of the domain and close to this is a conserved glutamine. A conserved tryptophan is located near the C-terminus, closely followed by two conserved hydrophobic amino acids. DUF3887 (Figure 2) contains a highly conserved glycine in the middle of the domain and two conserved hydrophobic amino acids near the C-terminus. DUF4878 (Figure 3) contains a conserved glycine about 25 amino acids into the domain and a conserved tryptophan near the C-terminus.

### Domain architectures

**Figure 4:**
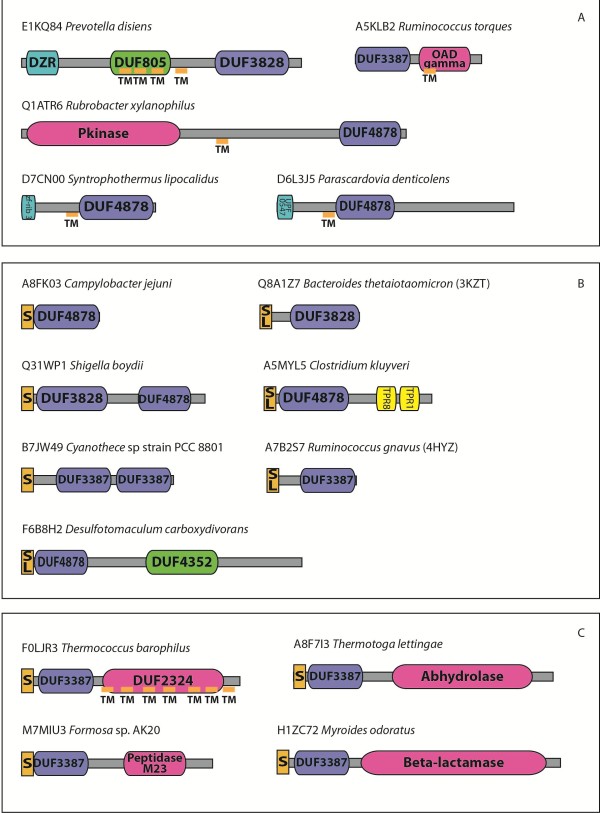
**Domain architectures of selected sequences containing NTF2-like domains.** Domain architectures were predicted by Pfam [12]. Signal peptides and transmembrane regions were predicted using Phobius [16]. The NTF2-liks DUFs are shown in blue, zinc ribbons in green, TPRs yellow, peptidases and other hydrolases in pink, other DUFs in bright green, and signal peptides (S), lipoboxes (SL) and transmembrane regions (TM) in yellow. Panel **A** shows architectures with extracellular ligand-sensing or intracellular signaling domains. Panel **B** shows secreted and lipid-anchored architectures. Panel **C** shows architectures including hydrolase domains.

**Figure 5:**
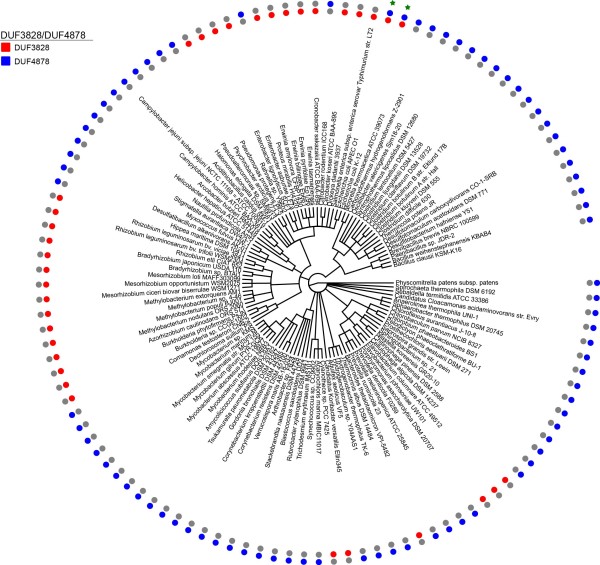
**Species distribution of DUF3828 and DUF4878.** Species tree was plotted using EvolView [14]. Red dots denote the presence of DUF3828 in a protein from the named species; blue dots denote the presence of DUF4878. Green stars indicate species that both domains are present in the species.

In Pfam, 224 of the 492 proteins (45%) containing DUF3828 also contain a DUF4878 domain at the C-terminus (Figure 4). Given the potential significance of this observation, we performed further investigation into the taxonomic distribution of these proteins. Using EvolView [14] we plotted a species tree containing members of the RP75 set of representative proteomes [15] which possess proteins containing DUF3828 and/or DUF4878 (Figure 5). Surprisingly, we found that only two of the 113 species in this tree possessed both domains, and therefore we conclude that the co-occurrence of these domains is not likely to be significant and is an artifact caused by the sequencing of a disproportionately large number of *Escherichia coli* strains compared to the other species these domains are found in.

Besides the apparent co-occurrence of DUF3828 and DUF4878, the three DUFs also occur in several other architectures in Pfam. These can be split roughly into three categories: Architectures suggestive of communication with extracellular ligand-sensing or intracellular signaling domains (Figure 4A), solo or multi-domain secreted and lipid-anchored architectures (Figure 4B) and fusions to C-terminal peptidase or other hydrolase domains (Figure 4C).

In the first category (Figure 4A), the intracellular domains to which the extrinsic DUF is linked include protein kinase domains and three distinct versions of zinc ribbons, which could potentially bind nucleic acids. These architectures are comparable to other signaling proteins where extracellular ligand domains are linked to intracellular signaling domains. This category also includes fusions of the DUF with the sodium pump associated oxaloacetate decarboxylase γ chain (OAD gamma). In the third category (Figure 4C) we observed independent fusions to metallopeptidase (M23), DUF2324 (a transmembrane domain which is a member of the Peptidase U clan), a beta-lactamase and an α/β hydrolase domain (Abhydrolase). In all of these cases the DUF is present at the N-terminus and the hydrolase domain at the C-terminus. NTF2-like domains have been observed with α/β hydrolases before: both SnoaL-like domain [Pfam:PF12680] and DUF4440 [Pfam:PF14534] co-occur with α/β hydrolase domains.

### Genomic context

We studied the genomic context of proteins containing DUF3828, DUF3887 and DUF4878. In doing this we hoped to glean information about the possible function of these domains. As a result we uncovered a conserved association with DUF3828, which in diverse gammaproteobacteria, betaprotebacteria and bacteroidetes is combined in an operon with a gene coding for a protein of the NlpC/P60 superfamily with a papain-like peptidase fold (e.g. gi: 489959630 from *Enterobacter cloacae*) [17]. These domains are known to function as peptidases/amidases in that cleave amide/peptide linkages in the bacterial cell wall. Several of these proteins additionally contain further C-terminal domains such as EF-hands, metallopeptidase family M23 and glycohydrolases of the lysozyme [Pfam:PF00959] or the Chitinase Class I [Pfam:PF00182] families. Thus, domains point to catalytic activities that process both the peptide and glycosidic linkages in peptidoglycan.

### Structure description

The crystal structure of a DUF3828 protein, BT_3511 protein [UniProtKB:Q8A1Z7] from *Bacteroides thetaiotaomicron* (strain VPI-5482), was determined to 2.1 Å resolution by MAD method and deposited to PDB as [PDB:3KZT]. The final model includes two molecules (residues 26–167), five 1,2-ethanediol, two sulfate ions and 118 water molecules in the asymmetric unit. The structure is mainly composed of three helices, one 3_10_ helix and 4 beta strands. Gly0 (that remained at the N-terminus after cleavage of the expression/purification tag), the region from Lys26 to Pro34 was disordered and not modeled. All the side chains were fully modeled because of the complete electron density. The Matthews coefficient (*V*_*M*_) is 2.05 Å^3^ Da^-1^ and the estimated solvent content is 39.97%. The Ramachandran plot produced by MolProbity [18] shows that 96.9% of the residues are in favored regions, with no outliers.

The crystal structure of a DUF4878 protein, Cj0202c protein [UniProtKB:Q0PBT7] from *Campylobacter jejuni* subsp. jejuni serotype O:2 (strain NCTC 11168), was determined to 2.0 Å resolution by MAD method and was deposited to PDB as [PDB:3K7C]. The final model includes four molecules (residues 1–113), one chloride ion, thirteen di hydroxyethyl ether (PEG), six triethylene glycol and 134 water molecules in the asymmetric unit. The structure is mainly composed of three helices and four beta strands. Gly0 (which remained at the N-terminus after cleavage of the expression/purification tag), the region from Met1 to Ser5 was disordered and not modeled. All the side chains were fully modeled because of the complete electron density. The Matthews coefficient (*V*_*M*_;) is 2.3 Å^3^ Da^-1^ and the estimated solvent content is 46.46%. The Ramachandran plot produced by MolProbity shows that 97.4% of the residues are in favoured regions, with no outliers.

The crystal structure of a DUF3887 protein, the hypothetical protein Rumgna_01855 [UniProtKB:A7B2S7] from *Ruminococcus gnavus* (strain ATCC 29149), was determined to 2.25 Å resolution by MAD method and was deposited to PDB as [PDB:4HYZ]. The final model includes two molecules (residues 36–149), six chloride ions, six sulfate ions, eight glycerol and 107 water molecules in the asymmetric unit. The structure is mainly composed of four helices, four turns, and five beta strands. Only Gly0 (that remained at the N-terminus after cleavage of the expression/purification tag) was disordered and not modeled. All the side chains were fully modeled because of the complete electron density. The Matthews coefficient (*V*_*M*_) is 3.00 Å^3^ Da^-1^ and the estimated solvent content is 58.98%. The Ramachandran plot produced by MolProbity shows that 99.6% of the residues are in favored regions, with no outliers.

Comparison of these three structures showed that they are significantly similar to each other, especially for [PDB:3K7C] and [PDB:4HYZ]. FATCAT results showed that the structures of [PDB:3KZT] and [PDB:4HYZ] are significantly similar with P-value of 1.53e-^03^ and the structure alignment has 85 equivalent positions with an RMSD of 1.72 Å; the structures of [PDB:3KZT] and [PDB:3K7C] are significantly similar with P-value of 2.57e-^03^ and the structure alignment has 95 equivalent positions with an RMSD of 2.53 Å; the structures of [PDB:4HYZ] and [PDB:3K7C] are significantly similar with P-value of 1.55e-^06^ and the structure alignment has 95 equivalent positions with an RMSD of 2.99 Å [19]. In all cases, FATCAT program detected flexibility in the structure, mostly limited to the relative position of helices with respect to the central beta sheet. There is low sequence similarity between the three structures, and no positions are conserved in all three structures.

**Figure 6:**
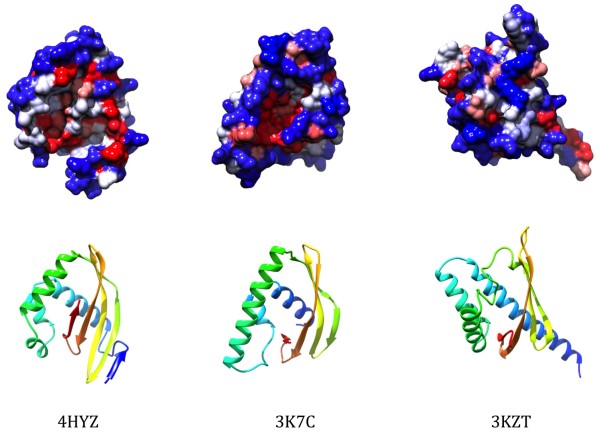
**Structures of representatives of DUF3828, DUF3887, and DUF4878.** [PDB:4HYZ] is a member of DUF3887, [PDB:3K7C] is a member of DUF4878 and [PDB:3KZT] is a member of DUF3828. Structures were aligned with POSA [22] and hydrophobic surface plots generated in Chimera [23].

**Figure 7:**
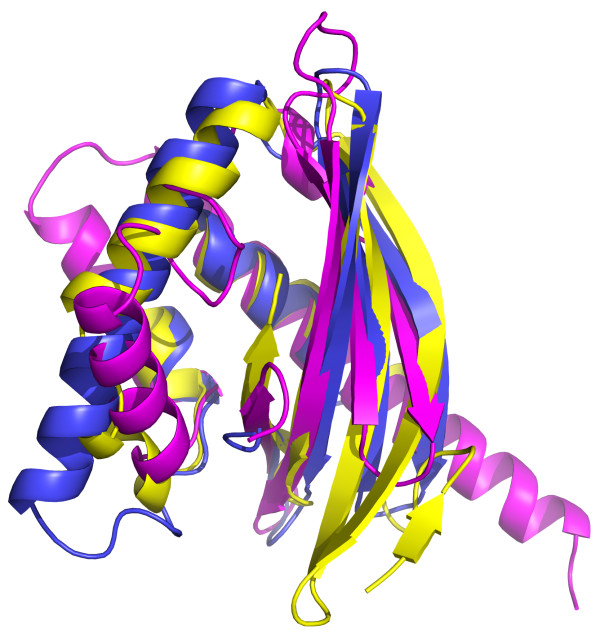
Structural superimposition of DUF3887 (blue), DUF4878 (yellow), and DUF3828 (magenta).

The three structures ([PDB:3K7C], [PDB:3KZT] and [PDB:4HYZ]) all possess NTF2-like folds, despite being dissimilar in sequence (Figures 6 and 7, Table 1). A hydrophobic cavity with the potential for ligand-binding has been described in NTF2-like proteins before [20]. We used the MarkUs functional annotation server to locate potential cavities within the three structures [21]. All three structures contain predicted cavities, but the position of these cavities is not conserved between the structures. The structure of [PDB:3K7C] differs from that of [PDB:3KZT] and [PDB:4HYZ] in that it lacks the edge strands in the beta-sheet but has a longer helix on the opposite side. It has a shallow cavity with positive electrostatic potential that contains a bound PEG molecule in the crystal structure. Notably, in dimers seen in the crystal structure of [PDB:3K7C] the cavities of individual subunits combine in a contiguous groove that can accommodate a larger ligand than a conventional NTF-like fold can. [PDB:4HYZ] has a cavity of a similar size in a similar position with weakly positive electrostatic potential. Sequence conservation in the region of the cavities in [PDB:3K7C] and in [PDB:4HYZ] is poor. In contrast, the cavity found in [PDB:3KZT] has a negative electrostatic potential, this cavity includes two highly conserved aspartic acid residues (D-103 and D-110) which may be of significance.

**Table 1:** **Percentage identity of the three proteins for which structure has been determined, calculated using DALI**[24]

**PDB ID**	**3KZT**	**3K7C**	**4HYZ**
**3KZT**		11%	14%
**3K7C**	11%		10%
**4HYZ**	14%	10%	

DALI [24] searches revealed that [PDB:3KZC] and [PDB:4HYZ] are more similar to each other than they are to most other members of the NTF2-like superfamily (Z-score 9.8), however [PDB:3KZT] is more distantly related. [PDB:3KZT] is most similar to [PDB:2UX0] (Z-score 8.8) which contains a Calcium/calmodulin dependent protein kinase II association domain [Pfam:PF08332], also a member of the NTF2-like superfamily. This domain functions as an oligomerisation domain [25]. It is also significantly similar to [PDB:2BHM] (Z-score 8.5), a member of the VirB8 family [Pfam:PF04335], a component of the type IV secretion system [10]. It is significantly less similar to [PDB:3K7C] (maximum Z-score of 6.4 when compared to chain A), and [PDB:4HYZ] (maximum Z-score of 6.8 when compared to chain B). [PDB:4HYZ] and [PDB:3K7C] are most similar to members of the SnoAL_3 family [Pfam:PF13474] including [PDB:3GWR] (Z-score 10.1 when compared to [PDB:3K7C]), and the SnoAL_2 family [Pfam:PF12860] including [PDB:3D9R] (Z-score 9.5 when compared to [PDB:3K7C]).

### Potential function

NTF2-like domains include both catalytic and non-catalytic versions that tend to bind small molecules using a common substrate-binding pocket. Our analysis of these DUFs did not reveal conserved polar residues suggestive of catalytic activity in DUF3887 or DUF4878. DUF3828 contains a conserved aspartic acid, which could point to a catalytic function. NTF2-like domains which are enzymatic tend to occur in an intracellular context, however prediction of subcellular localization using Phobius [16] revealed the consistent presence of either N-terminal secretory signals or lipoboxes with a conserved cysteine which helps anchor the protein to the membrane. Those proteins that lack either of these features have transmembrane regions with predicted membrane topologies suggestive of an extracellular location for the DUF (Figure 4). Together these observations suggested that these three DUFs are novel NTF2-like domains that are likely to be extracellular domains that recognize a small molecule ligand via their binding pocket.

Further evidence for such a function is offered by the domain architectures of these proteins (Figure 4). Where the DUF is found at the N-terminus of OAD γ chain domain the sensing of a ligand could help allosterically regulate sodium flux [26]. Where the DUF is found at the N-terminus of a protein containing a C-terminal peptidase or other hydrolase domain, it is conceivable that the sensing of a ligand by the N-terminal DUF regulates the catalytic domain. Similar domain architecture associations were also observed for DUF4352, which occurs fused to DUF4878 in certain contexts: DUF4352 is also linked to metallopeptidase (M56), protein kinase and TPR repeats and is also associated with lipid attachment signal or signal peptides or transmembrane regions. Hence it is possible that the two domains perform comparable functions and cooperate in recognition of extracellular ligands on occasions. The versions combined in operons with the NlpC/P60 like peptidases/amidases might potentially regulate the export and/or the activity of these peptidoglycan hydrolyzing proteins that could have a potentially suicidal effect on the cell. Thus, they could play a role in regulating peptidoglycan remodeling.

## Conclusions

Here we present a comparison of first crystal structures of three DUFs belonging to the NTF2-like superfamily. This work expands our structural knowledge of the sequence diverse NTF2 superfamily. Analysis of the three-dimensional structure, sequence and associated domains can provide clues about the likely function of a protein domain. We present a detailed analysis of these three domains, which suggests that they may play a role in binding to small molecule ligands.

## Methods

### Sequence and gene context analysis

Data for families DUF3828 and DUF4878 are taken from Pfam release 27.0 [12]. The definition of DUF3887 has been improved during the course of this work and the updated version will form a part of Pfam release 28.0. Signal peptides and transmembrane domains were predicted using Phobius [16]. A phylogenetic tree was constructed from proteomes in representative proteomes RP75 [15] using the NCBI taxonomy common tree [27]. This was annotated and displayed using EvolView [14].

With the DUF genes as anchors, the gene neighbourhood was also comprehensively analyzed using a custom Perl script. This script uses either the PTT file (downloadable from the NCBI ftp site) or the Genbank file in the case of whole genome shot gun sequences to extract the neighbors of a given query gene. The protein sequences of all neighbors were clustered using the BLASTCLUST program (ftp://ftp.ncbi.nih.gov/blast/documents/blastclust.html) to identify related sequences in gene neighbourhoods. Each cluster of homologous proteins were then assigned an annotation based on the domain architecture or conserved shared domain which were detected using Pfam models and in-house profiles run using RPS-BLAST [28]. This allowed an initial annotation of gene neighbourhoods and their grouping based on conservation of neighborhood associations. In further analysis care was taken to ensure that genes are unidirectional on the same strand of DNA and shared a putative common promoter to be counted as a single operon. If they were head to head on opposite strands they were examined for potential bidirection promoter sharing patterns.

### Structure determination

Protein purification and crystallization was performed by the JCSG crystallomics core [29-31]. All X-ray diffraction data were collected at the Stanford Synchrotron Radiation Lightsource (SSRL) on beamline 11–1. Data sets were collected at 100 K using a Rayonix MX-325 CCD detector. X-ray diffraction data were collected from a single crystal at wavelengths corresponding to the inflection (λ _1_), high energy remote (λ _2_), and peak (λ _3_) [PDB:3K7C]; the peak(λ _1_), inflection (λ _2_), and high energy remote (λ _3_) [PDB: 4HYZ]; or the inflection (λ _1_) and high energy remote (λ _2_) [PDB:3KZT], of a multi-wavelength or a two-wavelength selenium multi-wavelength anomalous diffraction (MAD). The data were integrated and scaled using the XDS and XSCALE programs respectively [32,33] [PDB:3K7C] or the MOSFLM [34] and SCALA [35] programs [PDB:3KZT][PDB:4HYZ]. Data statistics are summarized in Additional file 1: Tables S1-S3. The selenium substructures for the three proteins were solved with SHELXD [36] and the MAD phases were refined with autoSHARP [37]. Iterative automated model building was performed with RESOLVE [38] at a resolution of 2.00 Å [PDB:3K7C] or Arp/Warp [39] at a resolution of 2.15 Å [PDB:3KZT] or with Buccaneer [40,41] at a resolution of 2.25 Å [PDB:4HYZ] from density-modified electron density. Model completion was performed using the interactive computer-graphics program COOT [42] and MAD-phase-restrained refinement was accomplished using the program REFMAC ver 5.5.0102 [PDB:3K7C], ver 5.5.0053 [PDB:3KZT] [43] or BUSTER ver 2.10.0 [44] [PDB:4HYZ].

### Structure validation and deposition

The quality of the crystal structure was analyzed using the JCSG Quality Control Server [45]. This server verifies: the stereochemical quality of the model using AutoDepInputTool, MolProbity and WHATIF 5.0 [18,46,47]; agreement between the atomic model and the data using SFcheck 4.0 and RESOLVE [38,48]; the protein sequence using CLUSTALW [49]; atom occupancies using MOLEMAN2.0 [50]; and consistency of NCS pairs. It also evaluates differences in Rcryst/Rfree, expected Rfree/Rcryst, and maximum/minimum B-values by parsing the refinement log-file and PDB header. Protein quaternary structure analysis used the EBI PISA server [51]. Atomic coordinates and experimental structure factors have been deposited in the PDB and are accessible under the codes [PDB:3KZT], [PDB:3K7C] and [PDB:4HYZ]. Electrostatic potential and cavity prediction was performed using the MarkUs functional annotation server [21].

### Availabilty of supporting data

The data sets supporting the results of this article are included within the article (and its Additional file 1: Tables S1-S3).

## Competing interests

The authors declare that they have no competing interests.

## Authors’ contributions

RYE wrote the majority of the manuscript and produced Figures 1, 2, 3, 4 and 5. YC wrote a section of the manuscript and produced Figure 7. AB contributed to the study ideas and organization of the manuscript. AGM contributed to structure descriptions. HLA wrote structure determination and validation methods. WCH provided Figure 6. LA provided potential function discussion. All authors read and approved the final manuscript.

## Supplementary Material

Additional file 1: Table S1 Data collection and refinement statistics (PDB 3kzt). **Table S2.** Data collection and refinement statistics (PDB 3k7c). **Table S3.** Data collection and refinement statistics (PDB 4hyz). Click here for file
