# Supplementary material for: Filling out the structural map of the NTF2-like superfamily
Source: BMC Bioinformatics. 2013 Nov 19;14:327. doi: 10.1186/1471-2105-14-327 (PMC3924330; doi:10.1186/1471-2105-14-327)
Supplement: Additional file 1: Table S1 — Data collection and refinement statistics (PDB 3kzt). Table S2. Data collection and refinement statistics (PDB 3k7c). Table S3. Data collection and refinement statistics (PDB 4hyz). [file 1471-2105-14-327-S1.docx]

**Table S1. Data collection and refinement statistics (PDB 3kzt)**

| **Data collection** |  | | |
| --- | --- | --- | --- |
| Beamline | SSRL 11-1 | | |
| Space group and Unit cell | P2_1_2_1_2_1_, a=43.7, b=46.3, c=130.5 Å | | |
| Data | λ_1_ MADSe | λ_2_ MADSe |  |
| Wavelength (Å) | 0.97916 | 0.91837 |  |
| Resolution range (Å) | 28.47-2.15 | 28.6-2.10 |  |
| No. of observations | 51,273 | 54,689 |  |
| No. of unique reflections | 14,939 | 16,114 |  |
| Completeness (%) | 99.9 (100.0) | 99.9 (100.0) |  |
| Mean I/σ (I) | 9.8 (1.8) | 9.2 (1.6) |  |
| R_merge_ on I (%)^†^ | 10.6 (54.7) | 10.80 (63.0) |  |
| R_meas_ on I (%)^‡^ | 12.6 (64.5) | 12.80 (74.5) |  |
| R_pim_ on I (%)^‡‡^ | 6.7 (33.8) | 6.90 (39.4) |  |
| Highest resolution shell | 2.21-2.15 | 2.15-2.10 |  |
| **Model and refinement statistics** | | | |
| Data used in refinement | | λ_2_ MADSe |  |
| No. of reflections (total) | | 16,067^§^ |  |
| No. of reflections (test) | | 805 |  |
| Cutoff criteria | | \|F\|>0 |  |
| R_cryst_ (%)^¶^ | | 22.2 |  |
| R_free_ (%)^¶^ | | 26.8 |  |
| **Stereochemical parameters** | | | |
| Restraints (RMSD observed) | | | |
| Bond lengths (Å) | | 0.017 |  |
| Bond angles (°) | | 1.289 |  |
| MolProbity all atom clash score | | 3.16 |  |
| Ramachandran plot (%)^±^ | | 96.9 (0) |  |
| Rotamer outlier (%) | | 0 |  |
| Average isotropic B-value (Å^2^) ^††^ | | 18.4 |  |
| ESU based on R_free_ (Å) ^‡‡‡^ | | 0.224 |  |
| No. protein residues / chains | | 262/2 |  |
| Non-protein entities | | 4 EDO, 2 SO_4_, 118 H_2_O |  |

Values in parentheses are for the highest resolution shell.

^†^ *R_merge_* = Σ*_hkl_*Σ*_i_*|*I_i_(hkl) - (I(hkl))*|/Σ*_hkl_* Σ*_i_(hkl)*.

^‡^ *R_meas_* = Σ*_hkl_*[*N/(N*-1)]^1/2^Σ*_i_*|*I_i_(hkl) - (I(hkl))|/*Σ*_hkl_*Σ*_i_I_i_(hkl)*[[1](#_ENREF_1)].

^‡‡^ *R_p.i.m_* (precision-indicating *R_merge_*) = Σ*_hkl_*[(1/(*N*-1)] ^½^ Σ*_i_*|I*_i_* (*hkl*) - <I(*hkl*)>| / Σ*_hkl_*Σ*_i_* I*_i_*(*hkl*) [[2](#_ENREF_2), [3](#_ENREF_3)].

^§^ Typically, the number of unique reflections used in refinement is slightly less than the total number that were integrated and scaled. Reflections are excluded owing to negative intensities and rounding errors in the resolution limits and unit-cell parameters.

^¶^ *R_cryst_* = Σ*_hkl_*||*F*_obs_| - |*F*_calc_||/Σ*_hkl_*|*F*_obs_|, where *F*_calc_ and *F*_obs_ are the calculated and observed structure-factor amplitudes, respectively. *R_free_* is the same as *R_cryst_* but for 5.0% of the total reflections chosen at random and omitted from refinement.

^††^ This value represents the total *B* that includes TLS and residual *B* components.

^±^ Percentage of residues in favored regions of Ramachandran plot (No. outliers in parenthesis).

^‡‡‡^ Estimated overall coordinate error [[4](#_ENREF_4)].

**Table S2. Data collection and refinement statistics (PDB 3k7c)**

| **Data collection** |  | | |
| --- | --- | --- | --- |
| Beamline | SSRL 11-1 | | |
| Space group/Unit cell | C2, a=94.4, b=89.8,c=59.7 Å, β=110.5° | | |
| Data | λ_1_ MADSe | λ_2_ MADSe | λ_3_ MADSe |
| Wavelength (Å) | 0.97908 | 0.91837 | 0.97855 |
| Resolution range (Å) | 29.2-2.12 | 28.6-2.00 | 29.3-2.12 |
| No. of observations | 98,909 | 117,170 | 99,071 |
| No. of unique reflections | 26,412 | 31,219 | 26,419 |
| Completeness (%) | 99.4 (98.8) | 99.3 (99.0) | 99.4 (99.3) |
| Mean I/σ (I) | 14.4 (2.7) | 12.7 (1.6) | 14.1 (2.6) |
| R_merge_ on I (%)^†^ | 6.2 (50.4) | 7.0 (87.2) | 6.7 (54.1) |
| R_meas_ on I (%)^‡^ | 7.3 (59.0) | 8.2 (102.0) | 7.9 (63.2) |
| R_pim_ on I (%)^‡‡^ | 3.8 (30.3) | 4.2 (52.4) | 4.1 (32.4) |
| Highest resolution shell | 2.17-2.12 | 2.05-2.00 | 2.18-2.12 |
| **Model and refinement statistics** | | | |
| Data used in refinement | | λ_2_ MADSe |  |
| No. of reflections (total) | | 31,216 |  |
| No. of reflections (test) | | 1,576 |  |
| Cutoff criteria | | \|F\|>0 |  |
| R_cryst_ (%)^¶^ | | 21.4 |  |
| R_free_ (%)^¶^ | | 25.6 |  |
| **Stereochemical parameters** | | | |
| Restraints (RMSD observed) | | | |
| Bond lengths (Å) | | 0.017 |  |
| Bond angles (°) | | 1.73 |  |
| MolProbity all atom clash score | | 6.48 |  |
| Ramachandran plot (%)^±^ | | 97.6 (0) |  |
| Rotamer outlier (%) | | 1.1 |  |
| Average isotropic B-value (Å^2^) ^††^ | | 18.7 |  |
| ESU based on R_free_ (Å) ^‡‡‡^ | | 0.184 |  |
| No. protein residues / chains | | 432/4 |  |
| Non-protein entities | | 1 CL, 13 PEG, 6 PGE 134 H_2_O |  |

Values in parentheses are for the highest resolution shell.

^†^ *R_merge_* = Σ*_hkl_*Σ*_i_*|*I_i_(hkl) - (I(hkl))*|/Σ*_hkl_* Σ*_i_(hkl)*.

^‡^ *R_meas_* = Σ*_hkl_*[*N/(N*-1)]^1/2^Σ*_i_*|*I_i_(hkl) - (I(hkl))|/*Σ*_hkl_*Σ*_i_I_i_(hkl)*[[1](#_ENREF_1)].

^‡‡^ *R_p.i.m_* (precision-indicating *R_merge_*) = Σ*_hkl_*[(1/(*N*-1)] ^½^ Σ*_i_*|I*_i_* (*hkl*) - <I(*hkl*)>| / Σ*_hkl_*Σ*_i_* I*_i_*(*hkl*) [[2](#_ENREF_2), [3](#_ENREF_3)].

^§^ Typically, the number of unique reflections used in refinement is slightly less than the total number that were integrated and scaled. Reflections are excluded owing to negative intensities and rounding errors in the resolution limits and unit-cell parameters.

^¶^ *R_cryst_* = Σ*_hkl_*||*F*_obs_| - |*F*_calc_||/Σ*_hkl_*|*F*_obs_|, where *F*_calc_ and *F*_obs_ are the calculated and observed structure-factor amplitudes, respectively. *R_free_* is the same as *R_cryst_* but for 5.0% of the total reflections chosen at random and omitted from refinement.

^††^ This value represents the total *B* that includes TLS and residual *B* components.

^±^ Percentage of residues in favored regions of Ramachandran plot (No. outliers in parenthesis).

^‡‡‡^ Estimated overall coordinate error [[4](#_ENREF_4)].

**Table S3. Data collection and refinement statistics (PDB 4hyz)**

| **Data collection** |  | | |
| --- | --- | --- | --- |
| Beamline | SSRL 11-1 | | |
| Space group/Unit cell | P6_5_22, a=b=66.3, c=253.8 Å | | |
| Data | λ_1_ MADSe | λ_2_ MADSe | λ_3_ MADSe |
| Wavelength (Å) | 0.97871 | 0.97922 | 0.91837 |
| Resolution range (Å) | 29.4-2.25 | 29.3-2.37 | 29.4-2.31 |
| No. of observations | 206,680 | 173,596 | 167,388 |
| No. of unique reflections | 16,710 | 14,243 | 15,480 |
| Completeness (%) | 99.9 (99.9) | 99.9 (99.0) | 99.9 (100.0) |
| Mean I/σ (I) | 24.2 (2.6) | 17.5 (2.0) | 12.2 (1.6) |
| R_merge_ on I (%)^†^ | 9.3 (113.5) | 13.7 (170.9) | 16.8 (167.1) |
| R_meas_ on I (%)^‡^ | 9.7 (118.7) | 14.3 (177.9) | 17.7 (174.8) |
| R_pim_ on I (%)^‡‡^ | 2.7 (34.2) | 4.0 (49.0) | 5.3 (50.9) |
| Highest resolution shell | 2.31-2.25 | 2.44-2.37 | 2.44-2.31 |
| **Model and refinement statistics** | | | |
| Data used in refinement | | λ_1_ MADSe |  |
| No. of reflections (total) | | 16,601 |  |
| No. of reflections (test) | | 842 |  |
| Cutoff criteria | | \|F\|>0 |  |
| R_cryst_ (%)^¶^ | | 18.6 |  |
| R_free_ (%)^¶^ | | 20.8 |  |
| **Stereochemical parameters** | | | |
| Restraints (RMSD observed) | | | |
| Bond lengths (Å) | | 0.010 |  |
| Bond angles (°) | | 1.06 |  |
| MolProbity all atom clash score | | 0.8 |  |
| Ramachandran plot (%)^±^ | | 100 (0) |  |
| Rotamer outlier (%) | | 1.05 |  |
| Average isotropic B-value (Å^2^) ^††^ | | 51.5 |  |
| ESU based on R_free_ (Å) ^‡‡‡^ | | 0.163 |  |
| No. protein residues / chains | | 228/2 |  |
| Non-protein entities | | 6 SO_4_, 6 CL, 8 GOL, 107 H_2_O |  |

Values in parentheses are for the highest resolution shell.

^†^ *R_merge_* = Σ*_hkl_*Σ*_i_*|*I_i_(hkl) - (I(hkl))*|/Σ*_hkl_* Σ*_i_(hkl)*.

^‡^ *R_meas_* = Σ*_hkl_*[*N/(N*-1)]^1/2^Σ*_i_*|*I_i_(hkl) - (I(hkl))|/*Σ*_hkl_*Σ*_i_I_i_(hkl)*[[1](#_ENREF_1)].

^‡‡^ *R_p.i.m_* (precision-indicating *R_merge_*) = Σ*_hkl_*[(1/(*N*-1)] ^½^ Σ*_i_*|I*_i_* (*hkl*) - <I(*hkl*)>| / Σ*_hkl_*Σ*_i_* I*_i_*(*hkl*) [[2](#_ENREF_2), [3](#_ENREF_3)].

^§^ Typically, the number of unique reflections used in refinement is slightly less than the total number that were integrated and scaled. Reflections are excluded owing to negative intensities and rounding errors in the resolution limits and unit-cell parameters.

^¶^ *R_cryst_* = Σ*_hkl_*||*F*_obs_| - |*F*_calc_||/Σ*_hkl_*|*F*_obs_|, where *F*_calc_ and *F*_obs_ are the calculated and observed structure-factor amplitudes, respectively. *R_free_* is the same as *R_cryst_* but for 5.1% of the total reflections chosen at random and omitted from refinement.

^††^ This value represents the total *B* that includes TLS and residual *B* components.

^±^ Percentage of residues in favored regions of Ramachandran plot (No. outliers in parenthesis).

^‡‡‡^ Estimated overall coordinate error [[4](#_ENREF_4)].

**References**

1. Diederichs K, Karplus PA: **Improved R-factors for diffraction data analysis in macromolecular crystallography**. *Nature structural biology* 1997, **4**(4):269-275.

2. Weiss MS, Metzner HJ, Hilgenfeld R: **Two non-proline cis peptide bonds may be important for factor XIII function**. *FEBS Lett* 1998, **423**(3):291-296.

3. Weiss MS, Hilgenfeld R: **On the use of the merging R factor as a quality indicator for X-ray data**. *J Appl Crystallogr* 1997, **30**:203-205.

4. Cruickshank DW: **Remarks about protein structure precision**. *Acta Crystallogr D Biol Crystallogr* 1999, **55**(Pt 3):583-601.
